# Supplementary material for: Maternal weight change from prepregnancy to 18 months postpartum and subsequent risk of hypertension and cardiovascular disease in Danish women: A cohort study
Source: PLoS Med. 2021 Apr 2;18(4):e1003486. doi: 10.1371/journal.pmed.1003486 (PMC8051762; doi:10.1371/journal.pmed.1003486)
Supplement: S1 Table — GWG, gestational weight gain; IOM, Institute of Medicine. (DOCX) [file pmed.1003486.s002.docx]

| **S1 Table.** The 2009 Institute of Medicine´s (IOM) recommendations for gestational weight gain (GWG) according to prepregnancy BMI category | |
| --- | --- |
|  | GWG recommendation |
| Prepregnancy BMI (kg/m2) | kg |
| Underweight (<18.5) | 12.5-18 |
| Normal weight (18.5-24.9) | 11.5-16.0 |
| Overweight (25.0-29.9) | 7.0-11.5 |
| Obese (≥30) | 5.0-9.0 |
