# Supplementary material for: Maternal weight change from prepregnancy to 18 months postpartum and subsequent risk of hypertension and cardiovascular disease in Danish women: A cohort study
Source: PLoS Med. 2021 Apr 2;18(4):e1003486. doi: 10.1371/journal.pmed.1003486 (PMC8051762; doi:10.1371/journal.pmed.1003486)
Supplement: S2 Table — CI, confidence interval; HR, hazard ratio. (DOCX) [file pmed.1003486.s003.docx]

| **S2 Table.** Adjusted^a^ hazard ratios and rates (95^%^ Confidence Interval) of **self-reported hypertension** according to weight change from prepregnancy to 18 months postpartum, n=25,926 | | | | | |
| --- | --- | --- | --- | --- | --- |
|  | **Self-reported hypertension** | | | | |
|  | Cases | Adj. Rates^b^ | Adj. HR |  |  |
| **All** |  |  |  |  |  |
| <-1 | 366 | 42.4 | (35.1; 51.3) | 0.96 | (0.84; 1.09) |
| -1 to 1 | 823 | 44.2 | (37.4; 52.2) | Ref | |
| >1 to 2 | 263 | 56.1 | (46.3; 67.9) | 1.27 | (1.11; 1.46) |
| >2 | 171 | 59.4 | (48.1; 73.3) | 1.35 | (1.14; 1.59) |
| **BMI<25 kg/m2** |  |  |  |  |  |
| <-1 | 118 | 31.0 | (23.5; 41.1) | 0.93 | (0.76; 1.13) |
| -1 to 1 | 583 | 35.4 | (28.3; 44.1) | Ref | |
| >1 to 2 | 169 | 43.2 | (33.5; 55.6) | 1.24 | (1.04; 1.47) |
| >2 | 88 | 49.7 | (37.2; 66.6) | 1.46 | (1.16; 1.83) |
| **BMI≥25 kg/m2** |  |  |  |  |  |
| <-1 | 248 | 75.6 | (58.5; 97.6) | 0.96 | (0.78; 1.13) |
| -1 to 1 | 240 | 77.0 | (60.0; 99.4) | Ref | |
| >1 to 2 | 94 | 100.9 | (75.2; 135.5) | 1.33 | (1.01; 1.64) |
| >2 | 83 | 94.4 | (69.8; 127.8) | 1.22 | (0.91; 1.52) |
| ^a^ Adjusted for prepregnancy BMI, parity and alcohol intake before the index pregnancy, maternal age at conception, socio-occupational status, dietary intake, leisure-time exercise, diabetes, preeclampsia, and preterm birth during index pregnancy, smoking status during index pregnancy and the first 6 months postpartum, and total duration of breastfeeding | | | | | |
| ^b^ Reference woman: primiparous, 29.8 years of age at conception, prepregnancy BMI of 23.5 kg/m2 (for BMI<25 kg/m2 this was 21.5 kg/m2 and for BMI ≥25 kg/m2 this was 29.0 kg/m2), high in socio-occupational status, no preeclampsia, no diabetes, delivered at term, and during pregnancy was non-smoker, had an intermediate dietary pattern, did no exercise, and breastfed total 4 to 10 months. | | | | | |
