# Supplementary material for: Maternal weight change from prepregnancy to 18 months postpartum and subsequent risk of hypertension and cardiovascular disease in Danish women: A cohort study
Source: PLoS Med. 2021 Apr 2;18(4):e1003486. doi: 10.1371/journal.pmed.1003486 (PMC8051762; doi:10.1371/journal.pmed.1003486)
Supplement: S3 Table — CI, confidence interval; CVD, cardiovascular disease; HR, hazard ratio. (DOCX) [file pmed.1003486.s004.docx]

| **S3 Table.** Adjusted hazard ratios^a^ and rates^b^ (95% Confidence Interval) of hypertension and CVD according to weight change from prepregnancy to 18 months postpartum, n=27,645 – **complete case analyses** | | | | | | | | | | | | | |
| --- | --- | --- | --- | --- | --- | --- | --- | --- | --- | --- | --- | --- | --- |
|  | **Hypertension** | | | | | |  | **CVD** | | | | | |
|  | Cases (n) | Rate | 95% CI | HR | 95% CI | *P* value |  | Cases (n) | Rate | 95% CI | HR | 95% CI | *P* value |
| **All** |  |  |  |  |  |  |  |  |  |  |  |  |  |
| <-1 | 287 | 17.7 | (14.2, 21.9) | 0.98 | (0.84, 1.15) | 0.83 |  | 184 | 16.0 | (12.3, 20.8) | 1.10 | (0.91, 1.33) | 0.30 |
| -1 to 1 | 521 | 18.0 | (14.9, 21.7) | Ref | | |  | 375 | 14.5 | (11.5, 18.3) | Ref | | |
| >1 to 2 | 190 | 24.3 | (19.5, 30.2) | 1.35 | (1.15, 1.60) | <0.001 |  | 78 | 11.4 | (8.4, 15.4) | 0.78 | (0.61, 1.00) | 0.05 |
| >2 | 128 | 23.9 | (18.8, 30.5) | 1.34 | (1.10, 1.63) | 0.004 |  | 78 | 18.3 | (13.5, 24.7) | 1.26 | (0.98, 1.61) | 0.07 |
| **Prepregnancy BMI<25 kg/m^2^** | | | |  |  |  |  |  |  |  |  |  |  |
| <-1 | 86 | 14.6 | (10.5; 20.4) | 1.14 | (0.90, 1.45) | 0.29 |  | 88 | 17.9 | (12.6, 25.4) | 1.49 | (1.17, 1.91) | 0.001 |
| -1 to 1 | 314 | 13.4 | (10.2, 17.6) | Ref | | |  | 252 | 11.8 | (8.8, 15.9) | Ref | | |
| >1 to 2 | 109 | 18.2 | (13.3, 24.8) | 1.38 | (1.11, 1.72) | 0.004 |  | 51 | 9.6 | (6.5, 14.2) | 0.82 | (0.60, 1.10) | 0.18 |
| >2 | 56 | 18.9 | (13.2, 27.0) | 1.45 | (1.08, 1.92) | 0.012 |  | 45 | 17.1 | (11.5, 25.5) | 1.45 | (1.05, 1.99) | 0.02 |
| **Prepregnancy BMI≥25 kg/m^2^** | | | |  |  |  |  |  |  |  |  |  |  |
| <-1 | 201 | 31.3 | (23.8, 41.1) | 0.88 | (0.72, 1.07) | 0.20 |  | 96 | 17.9 | (12.2, 26.2) | 0.77 | (0.58, 1.00) | 0.05 |
| -1 to 1 | 207 | 35.6 | (27.2, 46.5) | Ref | | |  | 123 | 23.6 | (16.4, 34.0) | Ref | | |
| >1 to 2 | 81 | 46.1 | (33.7, 63.1) | 1.31 | (1.01, 1.69) | 0.04 |  | 27 | 16.6 | (10.1, 27.1) | 0.70 | (0.46, 1.06) | 0.09 |
| >2 | 72 | 42.0 | (30.4; 58.0) | 1.21 | (0.92, 1.58) | 0.17 |  | 33 | 23.0 | (14.5, 36.5) | 0.96 | (0.65, 1.41) | 0.82 |
| CVD: cardiovascular disease (ischemic heart disease and stroke) | | | | | | | | | | | | | |
| ^a^ Cox regression models were used to estimate hazard ratios and 95% confidence intervals adjusted for prepregnancy BMI, parity and alcohol intake before the index pregnancy, maternal age at conception, socio-occupational status, dietary intake, leisure-time exercise, diabetes, preeclampsia, and preterm birth during index pregnancy, smoking status during index pregnancy and the first 6 months postpartum, and total duration of breastfeeding | | | | | | | | | | | | | |
| ^b^ Poisson regression models were used to estimate rates and 95% confidence intervals per 10,000 person-years for a reference woman: primiparous, 29.8 years of age at conception, prepregnancy BMI of 23.5 kg/m2 (for BMI<25 kg/m2 this was 21.5 kg/m2 and for BMI ≥25 kg/m2 this was 29.0 kg/m2), high in socio-occupational status, no preeclampsia, no diabetes, delivered at term, and during pregnancy was non-smoker, had an intermediate dietary pattern, did no exercise, and breastfed total 4 to 10 months. | | | | | | | | | | | | | |
