# Supplementary material for: Maternal weight change from prepregnancy to 18 months postpartum and subsequent risk of hypertension and cardiovascular disease in Danish women: A cohort study
Source: PLoS Med. 2021 Apr 2;18(4):e1003486. doi: 10.1371/journal.pmed.1003486 (PMC8051762; doi:10.1371/journal.pmed.1003486)
Supplement: S4 Table — CI, confidence interval; CVD, cardiovascular disease; GWG, gestational weight gain; HR, hazard ratio; IOM, Institute of Medicine. (DOCX) [file pmed.1003486.s005.docx]

| **S4 Table.** Adjusted hazard ratios^a^ (95% Confidence Interval) of hypertension and CVD according to adherence to the IOM recommendations for gestational weight gain (GWG) and weight change from prepregnancy to 18 months postpartum, n=27,449 – **complete case analyses** | | | | | | | | | | | | | | | | | | | |
| --- | --- | --- | --- | --- | --- | --- | --- | --- | --- | --- | --- | --- | --- | --- | --- | --- | --- | --- | --- |
|  | **Hypertension** | | | | | | | | |  | **CVD** | | | | | | | | |
|  | GWG recommendations | | | | | | | | |  | GWG recommendations | | | | | | | | |
|  | Below | | | Within | | | Above | | |  | Below | | | Within | | | Above | | |
| Weight change prepregnancy to 18 months postpartum (BMI units) | HR | 95% CI | *P* value | HR | 95% CI | *P* value | HR | 95% CI | *P* value |  | HR | 95% CI | *P* value | HR | 95% CI | *P* value | HR | 95% CI | *P* value |
| **All** | n=4,732 | | | n=10,336 | | | n=12,381 | | |  | n=4,732 | |  | n=10,336 | |  | n=12,381 | |  |
| <-1 | 1.11 | (0.83, 1.47) | 0.48 | 1.07 | (0.83, 1.38) | 0.61 | 0.94 | (0.74, 1.19) | 0.60 |  | 1.27 | (0.91, 1.78) | 0.15 | 0.96 | (0.70, 1.33) | 0.33 | 1.12 | (0.84, 1.49) | 0.43 |
| -1 to 1 | 1.06 | (0.82, 1.36) | 0.66 | Ref | | | 1.08 | (0.89, 1.31) | 0.42 |  | 0.86 | (0.64, 1.16) | 0.33 | Ref | |  | 1.06 | (0.85, 1.33) | 0.60 |
| >1 | 1.34 | (0.90, 2.00) | 0.15 | 1.56 | (1.22, 1.99) | 0.002 | 1.36 | (1.11, 1.67) | 0.003 |  | 0.69 | (0.38, 1.25) | 0.22 | 0.65 | (0.44, 0.96) | 0.03 | 1.18 | (0.92, 1.51) | 0.21 |
| **Prepregnancy BMI<25 kg/m^2^** | n=4,000 | | | n=8,520 | | | n=7,361 | | |  | n=4,000 | |  | n=8,520 | |  | n=7,361 | |  |
| <-1 | 1.13 | (0.73, 1.75) | 0.57 | 0.95 | (0.63, 1.45) | 0.81 | 1.47 | (0.99, 2.18) | 0.06 |  | 1.96 | (1.33, 2.89) | 0.001 | 1.13 | (0.73, 1.75) | 0.59 | 1.62 | (1.06, 2.48) | 0.03 |
| -1 to 1 | 1.21 | (0.91, 1.61) | 0.19 | Ref | |  | 1.11 | (0.86, 1.43) | 0.44 |  | 0.98 | (0.71, 1.36) | 0.92 | Ref | |  | 1.03 | (0.78, 1.38) | 0.82 |
| >1 | 1.50 | (0.95, 2.36) | 0.08 | 1.58 | (1.17, 2.14) | 0.003 | 1.44 | (1.08, 1.91) | 0.01 |  | 0.62 | (0.30, 1.27) | 0.19 | 0.76 | (0.49, 1.18) | 0.23 | 1.35 | (0.98, 1.85) | 0.06 |
| **Prepregnancy BMI≥25 kg/m^2^** | n=732 | | | n=1,816 | | | n=5,020 | | |  | n=732 | |  | n=1,816 | |  | n=5,020 | |  |
| <-1 | 0.93 | (0.63, 1.38) | 0.73 | 0.93 | (0.65, 1.33) | 0.70 | 0.66 | (0.47, 0.93) | 0.02 |  | 0.53 | (0.29, 0.97) | 0.04 | 0.62 | (0.38, 1.02) | 0.06 | 0.68 | (0.44, 1.03) | 0.07 |
| -1 to 1 | 0.75 | (0.45, 1.25) | 0.27 | Ref | |  | 0.90 | (0.66, 1.23) | 0.49 |  | 0.52 | (0.24, 1.12) | 0.10 | Ref | |  | 0.85 | (0.57, 1.26) | 0.42 |
| >1 | 0.94 | (0.40, 2.18) | 0.88 | 1.39 | (0.90, 2.13) | 0.13 | 1.08 | (0.78, 1.49) | 0.65 |  | 1.05 | (0.38, 2.97) | 0.56 | 0.92 | (0.16, 0.88) | 0.03 | 0.76 | (0.50, 1.17) | 0.21 |
| CVD: cardiovascular disease (ischemic heart disease and stroke) | | | | | | | | | | | | | | | | | | | |
| ^a^ Cox regression models were used to estimate hazard ratios and 95% confidence intervals adjusted for prepregnancy BMI, parity and alcohol intake before the index pregnancy, maternal age at conception, socio-occupational status, dietary intake, leisure-time exercise, diabetes, preeclampsia, and preterm birth during index pregnancy, smoking status during index pregnancy and the first 6 months postpartum, and total duration of breastfeeding | | | | | | | | | | | | | | | | | | | |
