# Supplementary material for: Maternal weight change from prepregnancy to 18 months postpartum and subsequent risk of hypertension and cardiovascular disease in Danish women: A cohort study
Source: PLoS Med. 2021 Apr 2;18(4):e1003486. doi: 10.1371/journal.pmed.1003486 (PMC8051762; doi:10.1371/journal.pmed.1003486)
Supplement: S5 Table — CI, confidence interval; CVD, cardiovascular disease; HR, hazard ratio. (DOCX) [file pmed.1003486.s006.docx]

| **S5 Table.** Adjusted hazard ratios ^a^ (95% CI) of hypertension and CVD according to weight change patterns from prepregnancy to18 months postpartum, n=27,230 – **complete case analyses** | | | | | | | | | |
| --- | --- | --- | --- | --- | --- | --- | --- | --- | --- |
|  |  |  | **Hypertension** | | |  | **CVD** | | |
| Weight change pre-pregnancy to 18 months postpartum (BMI units) | Postpartum weight change pattern^b^ | n | HR | 95% CI | *P* value |  | HR | 95% CI | *P* value |
| **All** | | |  |  |  |  |  |  |  |
| <-1 | Early loss | 778 | 1.46 | (1.12, 1.92) | 0.006 |  | 1.36 | (0.94, 1.97) | 0.10 |
|  | Late loss | 4,738 | 0.91 | (0.77, 1.08) | 0.27 |  | 1.07 | (0.87, 1.31) | 0.51 |
| -1 to 1 | Stable | 15,687 | Ref | | |  | Ref | | |
| >1 | Retention | 3,024 | 1.30 | (1.08, 1.57) | 0.005 |  | 1.01 | (0.79, 1.29) | 0.91 |
|  | New gain | 3,003 | 1.41 | (1.18, 1.68) | <0.001 |  | 0.95 | (0.74, 1.22) | 0.67 |
| **Prepregnancy BMI<25 kg/m^2^** | | |  |  |  |  |  |  |  |
| <-1 | Early loss | 355 | 1.75 | (1.08, 2.86) | 0.02 |  | 1.50 | (0.82, 2.74) | 0.19 |
|  | Late loss | 2,379 | 1.05 | (0.80, 1.37) | 0.73 |  | 1.51 | (1.17, 1.96) | 0.002 |
| -1 to 1 | Stable | 12,751 | Ref | | |  | Ref | | |
| >1 | Retention | 2,298 | 1.31 | (1.02, 1.68) | 0.03 |  | 1.07 | (0.79, 1.44) | 0.67 |
|  | New gain | 2,071 | 1.47 | (1.15, 1.88) | 0.002 |  | 1.04 | (0.76, 1.44) | 0.79 |
| **Prepregnancy BMI≥25 kg/m^2^** | | |  |  |  |  |  |  |  |
| <-1 | Early loss | 423 | 1.32 | (0.95, 1.82) | 0.10 |  | 1.14 | (0.71, 1.82) | 0.59 |
|  | Late loss | 2,359 | 0.81 | (0.66, 1.01) | 0.10 |  | 0.70 | (0.52, 0.94) | 0.02 |
| -1 to 1 | Stable | 2,936 | Ref | | |  | Ref | | |
| >1 | Retention | 726 | 1.28 | (0.96, 1.70) | 0.09 |  | 0.90 | (0.59, 1.36) | 0.61 |
|  | New gain | 932 | 1.30 | (1.01, 1.68) | 0.04 |  | 0.76 | (0.50, 1.13) | 0.18 |
| CVD: cardiovascular disease (ischemic heart disease and stroke) | | | | | | | | | |
| ^a^ Cox regression models were used to estimate hazard ratios and 95% confidence intervals adjusted for prepregnancy BMI, parity and alcohol intake before the index pregnancy, maternal age at conception, socio-occupational status, dietary intake, leisure-time exercise, diabetes, preeclampsia, and preterm birth during index pregnancy, smoking status during index pregnancy and the first 6 months postpartum, and total duration of breastfeeding | | | | | | | | | |
| ^b^ Indicate how the overall weight change from prepregnancy to 18 months postpartum was reached by including weight 6 months postpartum. Early loss: 6 months weight<18 months weight, Late loss: 6 months weight≥18 months weight, New gain: 6 months weight<18 months weight, and Retention: 6 months weight≥18 months weight | | | | | | | | | |
