# Supplementary material for: Maternal weight change from prepregnancy to 18 months postpartum and subsequent risk of hypertension and cardiovascular disease in Danish women: A cohort study
Source: PLoS Med. 2021 Apr 2;18(4):e1003486. doi: 10.1371/journal.pmed.1003486 (PMC8051762; doi:10.1371/journal.pmed.1003486)
Supplement: S6 Table — CI, confidence interval; CVD, cardiovascular disease; HR, hazard ratio. (DOCX) [file pmed.1003486.s007.docx]

| **S6 Table.** Adjusted sub-distribution hazard ratios^a^ (95% Confidence Interval) of hypertension and CVD according to weight change from prepregnancy to 18 months postpartum, n=27,645 – **complete case analyses** | | | | | | | | | | |
| --- | --- | --- | --- | --- | --- | --- | --- | --- | --- | --- |
|  | **Hypertension** | | | |  | **CVD** | | | | |
|  | Cases (n) | HR | 95% CI | *P* value |  | Cases (n) | HR | | 95% CI | *P* value |
| **All** |  |  |  |  |  |  |  | |  |  |
| <-1 | 287 | 0.98 | (0.84, 1.15) | 0.84 |  | 184 | 1.06 | (0.91, 1.34) | | 0.31 |
| -1 to 1 | 521 | Ref | | |  | 375 | Ref | | | |
| >1 to 2 | 190 | 1.36 | (1.15, 1.60) | <0.001 |  | 78 | 0.78 | (0.61, 1.00) | | 0.05 |
| >2 | 128 | 1.34 | (1.10, 1.63) | 0.004 |  | 78 | 1.26 | (0.98, 1.62) | | 0.07 |
| **Prepregnancy BMI<25 kg/m^2^** | | | | | | | | | | |
| <-1 | 86 | 1.14 | (0.90, 1.45) | 0.28 |  | 88 | 1.49 | (1.16, 1.91) | | 0.002 |
| -1 to 1 | 314 | Ref | | |  | 252 | Ref | | | |
| >1 to 2 | 109 | 1.38 | (1.11, 1.71) | 0.004 |  | 51 | 0.81 | (0.60, 1.10) | | 0.18 |
| >2 | 56 | 1.45 | (1.09, 1.98) | 0.01 |  | 45 | 1.45 | (1.05, 2.01) | | 0.02 |
| **Prepregnancy BMI≥25 kg/m^2^** | | | | | | | | | | |
| <-1 | 201 | 0.88 | (0.72, 1.07) | 0.21 |  | 96 | 0.77 | (0.55, 1.01) | | 0.06 |
| -1 to 1 | 207 | Ref | | |  | 123 | Ref | | | |
| >1 to 2 | 81 | 1.32 | (1.02, 1.70) | 0.04 |  | 27 | 0.70 | (0.46, 1.07) | | 0.10 |
| >2 | 72 | 1.21 | (0.93, 1.59) | 0.16 |  | 33 | 0.96 | (0.65, 1.41) | | 0.84 |
| CVD: cardiovascular disease (ischemic heart disease and stroke) | | | | | | | | | | |
| ^a^ Fine – Gray method was used to estimate sub-distribution hazard ratios and 95% confidence intervals adjusted for prepregnancy BMI, parity and alcohol intake before the index pregnancy, maternal age at conception, socio-occupational status, dietary intake, leisure-time exercise, diabetes, preeclampsia, and preterm birth during index pregnancy, smoking status during index pregnancy and the first 6 months postpartum, and total duration of breastfeeding | | | | | | | | | | |
