# Supplementary material for: Maternal weight change from prepregnancy to 18 months postpartum and subsequent risk of hypertension and cardiovascular disease in Danish women: A cohort study
Source: PLoS Med. 2021 Apr 2;18(4):e1003486. doi: 10.1371/journal.pmed.1003486 (PMC8051762; doi:10.1371/journal.pmed.1003486)
Supplement: S1 Fig — Adjusted HRs (95% CI) of hypertension (A) and CVD (B) in relation to weight change from prepregnancy to 18 months postpartum in BMI units (all women; women with a prepregnancy BMI <25; and women with a prepregnancy BMI ≥25). Adjusted for maternal age at conception, socio-occupational status, parity, prepregnancy BMI, alcohol intake before the index pregnancy and dietary intake, leisure-time exercise, diabetes, preeclampsia, and preterm birth during index pregnancy, smoking status during index pregnancy and the first 6 months postpartum, and total duration of breastfeeding. CI, confidence interval; CVD, cardiovascular disease; HR, hazard ratio. (DOCX) [file pmed.1003486.s008.docx]

**
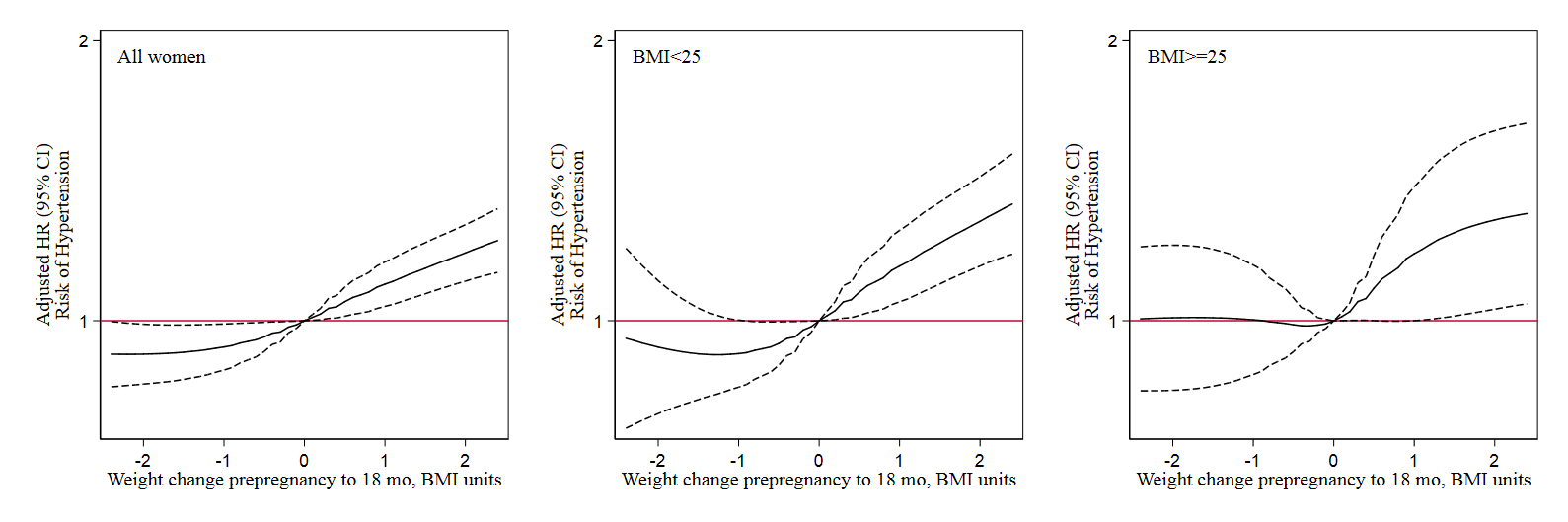
**

**A**

**
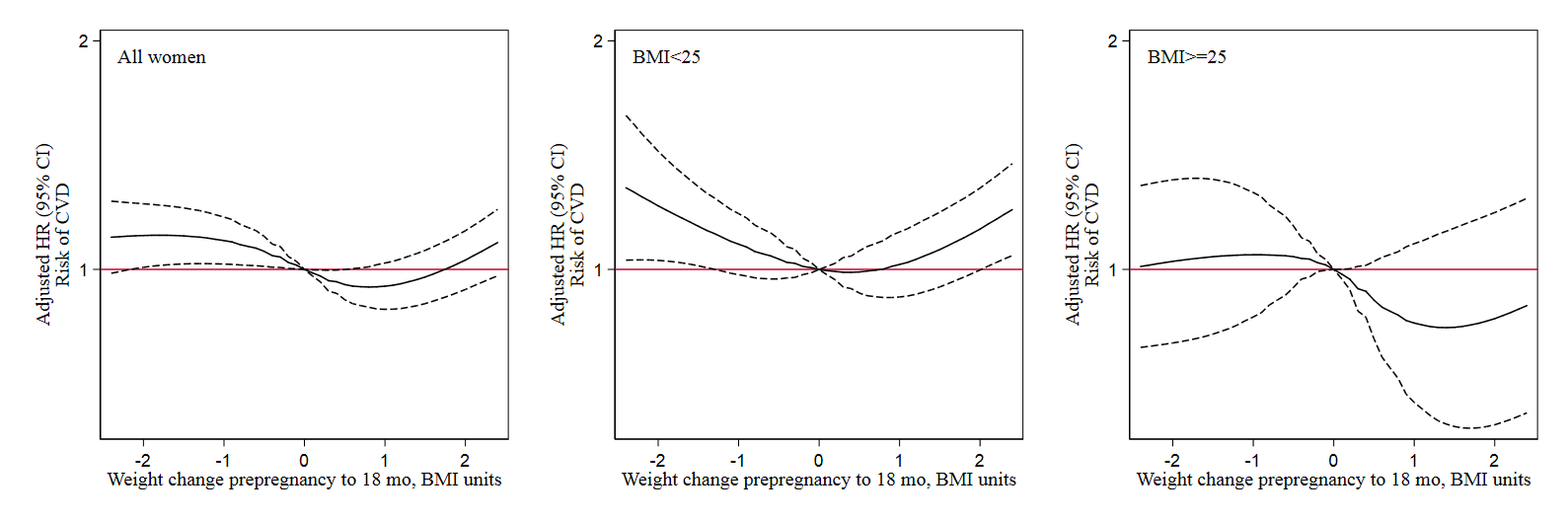
**

**B**

**S1 Fig** Adjusted hazard ratios (HR) (95% confidence interval) of hypertension (A) and cardiovascular disease (CVD) (B) in relation to weight change from prepregnancy to 18 months postpartum in BMI units (All women; women with a prepregnancy BMI <25; and women with a prepregnancy BMI ≥25). Adjusted for maternal age at conception, socio-occupational status, parity, prepregnancy BMI, alcohol intake before the index pregnancy, and dietary intake, leisure-time exercise, diabetes, preeclampsia, and preterm birth during index pregnancy, smoking status during index pregnancy and the first 6 months postpartum, and total duration of breastfeeding.
