# Supplementary material for: Maternal weight change from prepregnancy to 18 months postpartum and subsequent risk of hypertension and cardiovascular disease in Danish women: A cohort study
Source: PLoS Med. 2021 Apr 2;18(4):e1003486. doi: 10.1371/journal.pmed.1003486 (PMC8051762; doi:10.1371/journal.pmed.1003486)
Supplement: Attachment — Submitted filename: Comments to the authors 2020-04-01.docx [file pmed.1003486.s009.docx]

## Comments to the authors

This study investigates maternal weight change from pre-pregnancy to 18 months postpartum and subsequent risk of hypertension and cardiovascular disease.

This study is well-written, clearly described, include relevant tables and figures, and are of highly clinical relevance. Hence, I have very few comments. Although the self-reported weights are a limitation (as acknowledged by the authors) the fact that the authors have access to 18 months postpartum weights are a great strength that balance up the limitations of the self-reported weights.

As I understand from the submission system this article has already undergone one round of revision (?) but as I was not involved in the first round nor have seen the other reviewers’ comments, my comments are totally new and not related to any previous comments.

1. **Missing data:** In the method section the authors describe that they are using multiple imputation for imputation of covariates with missing values. However, they are not imputed the values for those with missing pre-pregnancy BMI as these instead are excluded from the cohort, why are not the values of the 5743 missing BMI (i.e. corresponding to 8% missing) imputed?
2. **Association of losing weight and increased risk of CVD among women with a BMI <25**

The study concludes that the women with a BMI<25 that have a weight loss of 1 unit in BMI from pre-pregnancy until 18 mo postpartum may have an increased risk of CVD, and this association foremost seen in the group of women that also gain below the IOM guidelines in weight during pregnancy.

The mechanisms are somewhat discussed in the discussion, but I wonder if the authors have further looked into this association and tried to find an explanation? Are the numbers sufficient to stratify BMI further, i.e. separate underweight and normal weight – could it be that this relationship could be modified by BMI further, i.e. is there a stronger relationship among underweight for example. There could also be unmeasured confounding, which the authors also discussed somewhat. Further, as the follow-up time is 16 years there could also be other factors /weight loss/wegiht gain during this period of time that could play an important role. Perhaps this could be discussed even further in the discussion.

**Minor**

Abstract – New weight gain is a bit hard to understand when reading only the abstract, hence adding the time-period to define new gainers would be beneficial

Figure 2 – This figure is beautiful and clearly describes the different weight patterns, however it would benefit much by adding different colors to the lines.
