# Supplementary material for: Maternal weight change from prepregnancy to 18 months postpartum and subsequent risk of hypertension and cardiovascular disease in Danish women: A cohort study
Source: PLoS Med. 2021 Apr 2;18(4):e1003486. doi: 10.1371/journal.pmed.1003486 (PMC8051762; doi:10.1371/journal.pmed.1003486)
Supplement: Attachment — Submitted filename: Kirkegaard_Response to reviewers_20200702.docx [file pmed.1003486.s010.docx]

**"Maternal weight change from prepregnancy to 18 months postpartum and subsequent risk of hypertension and cardiovascular disease: A study within the Danish National Birth Cohort" (PMEDICINE-D-19-04657R1)**

**Requests from the editors:**

Abstract

* Please structure your abstract using the PLOS Medicine headings (Background, Methods and Findings, Conclusions).

* Please combine the Methods and Findings sections into one section, “Methods and findings”.

* Please include the study design, population and setting, number of participants, years during which the study took place, length of follow up, and main outcome measures.

* Please quantify the main results (with 95% CIs and p values).

*We have revised the abstract accordingly. All main results already include 95% CI and we have added p values.*

* In the last sentence of the Abstract Methods and Findings section, please describe the main limitation(s) of the study's methodology.

*We have added a sentence to section about the main limitation in this study.*

References- in square brackets and bibliography formatting in Vancouver style

*We have revised this accordingly.*

At this stage, we ask that you include a short, non-technical Author Summary of your research to make findings accessible to a wide audience that includes both scientists and non-scientists. The Author Summary should immediately follow the Abstract in your revised manuscript. This text is subject to editorial change and should be distinct from the scientific abstract. Please see our author guidelines for more information: <https://journals.plos.org/plosmedicine/s/revising-your-manuscript#loc-author-summary>

*We have added this Author Summary to the manuscript.*

***Why Was This Study Done?***

- *Many women experience persistent weight gain from childbearing. This pregnancy-related weight change may be associated with worse long-term cardiovascular health.*

***What Did the Researchers Do and Find?***

- *We used data from 47,966 mothers who participated in the Danish National Birth Cohort.*
- *Self-reported weights were used to define their weight change patterns from prepregnancy to 6 and 18 months postpartum. We examined how these patterns were related to their risk of hypertension and cardiovascular disease the following 16 years.*
- *We found that with weight gain from before pregnancy to 18 months postpartum was positively associated with the risk of hypertension regardless of whether the women retained weight from pregnancy or gained weight from 6-18 months postpartum.*
- *In normal/underweight women, the risk of cardiovascular disease also increased in those who lost weight before pregnancy to 18 months postpartum, especially if they had gained below recommended during pregnancy. No such increased risks were observed in overweight/obese women.*

***What Do These Findings Mean?***

- *Our findings suggest that health professionals should also focus on the mother’s weight change patterns after given birth to improve their cardiovascular health. Although overweight women should avoid weight gain, both weight gain and loss should be of concern among normal/underweight women.*

Please provide citations to, or copies of questionnaires and interview guides used in the study

*In the method section, we have added a link to the webpage of the cohort where all questionnaires are available.*

*Line 83-84: “All questionnaires are available at* [*https://www.dnbc.dk/data-available*](https://www.dnbc.dk/data-available)*.”*

Did your study have a prospective protocol or analysis plan? Please state this (either way) early in the Methods section. a) If a prospective analysis plan (from your funding proposal, IRB or other ethics committee submission, study protocol, or other planning document written before analyzing the data) was used in designing the study, please include the relevant prospectively written document with your revised manuscript as a Supporting Information file to be published alongside your study, and cite it in the Methods section. A legend for this file should be included at the end of your manuscript. b) If no such document exists, please make sure that the Methods section transparently describes when analyses were planned, and when/why any data-driven changes to analyses took place. c) In either case, changes in the analysis-- including those made in response to peer review comments-- should be identified as such in the Methods section of the paper, with rationale.

*This study was developed from part of a previous PhD-project and we do not have a specific protocol for it. The initial study both focused on weight change and breastfeeding as predictors of CVD and was clearly overloaded with information and results. However, the hypothesis and analyses were planned a priori as part of the design of this specific study. Thus, is now stated in the beginning of the statistical analyses section.*

*Line 155: “All analyses were planned a priori when this study was designed.”*

Please ensure that the study is reported according to the [STROBE] guideline, and include the completed [STROBE or other] checklist as Supporting Information. When completing the checklist, please use section and paragraph numbers, rather than page numbers. Please add the following statement, or similar, to the Methods: "This study is reported as per the Strengthening the Reporting of Observational Studies in Epidemiology (STROBE) guideline (S1 Checklist)."

*We have updated the already submitted STROBE checklist and added the statement to the method section (line 99-100).*

On Page 9, second paragraph you have included an instance of “data not shown”, which is not permitted as per PLOS data policy. Please remove this instance or include the data as main text or SI files.

*We have now removed this instance from the manuscript (line 180).*

On page 10, please introduce the term GDM on first view

*We have introduced the term GDM by writing gestational diabetes mellitus (GDM) here in the manuscript (line 208).*

Please provide 95% CI and p values whenever reporting numerical results or as needed

*We had already presented all numerical estimates of association with 95% CI and now we have added p-values as well. For the test of possible effect modification of the associations by prepregnancy BMI, we have presented p-values.*

Page 11 last paragraph states “GWG recommendation had 70% (24-235%)”. Please correct and clarify as needed

*We have corrected to 70% (24-135%) here and in the abstract (line 20 and 244).*

Please present and organize the Discussion as follows: a short, clear summary of the article's findings; what the study adds to existing research and where and why the results may differ from previous research; strengths and limitations of the study; implications and next steps for research, clinical practice, and/or public policy; one-paragraph conclusion.

*We have organized the discussion as described and added a part about implications and next steps for research.*

*Line 355-361: “Future studies are needed that include information on maternal prepregnancy metabolic status and weight history which will help us to elucidate the specific influence of postpartum weight change on later cardiovascular health. However, our findings suggest mother´s postpartum weight changes also influence her long-term risk of hypertension and cardiovascular diseases. Thus, our findings support the concept that health care providers should expand their existing focus on pregnant women’s weight to include postpartum weight.”*

**Comments from the reviewers:**

**Reviewer #1:**

Comments to the authors

This study investigates maternal weight change from pre-pregnancy to 18 months postpartum and subsequent risk of hypertension and cardiovascular disease.

This study is well-written, clearly described, include relevant tables and figures, and are of highly clinical relevance. Hence, I have very few comments.

*Thanks a lot.*

Although the self-reported weights are a limitation (as acknowledged by the authors) the fact that the authors have access to 18 months postpartum weights are a great strength that balance up the limitations of the self-reported weights.

*Thanks, again. The 6- and 18-months postpartum weights are now mentioned explicitly as a strength in the discussion section.*

*Line 326-330: “The strengths of our study include our ability to study maternal weight changes by using prospectively collected maternal report of weights which reduces risk of recall bias. Further, the repeated weight measures, including both 6 and 18 months postpartum, made it possible to study different weight patterns, which is important as women may experience very different weight patterns in pregnancy and early motherhood.”*

As I understand from the submission system this article has already undergone one round of revision (?) but as I was not involved in the first round nor have seen the other reviewers' comments, my comments are totally new and not related to any previous comments.

*This is the first round of revision of this manuscript.*

1.	Missing data: In the method section the authors describe that they are using multiple imputation for imputation of covariates with missing values. However, they are not imputed the values for those with missing pre-pregnancy BMI as these instead are excluded from the cohort, why are not the values of the 5743 missing BMI (i.e. corresponding to 8% missing) imputed?

*We did not impute prepregnancy BMI, as we avoided imputing our main exposure, weight change from prepregnancy to 18 months postpartum. Also, 55% of those missing prepregnancy BMI are also missing data on weight at 18 months postpartum and would have been excluded anyway. Further, the more we increase missingness of data in the study population, the more our analyses depend on the assumption that data are missing at random and is subject to the increase in variance by imputation. This may be a limitation of our study related to generalizability of the findings.*

2.	Association of losing weight and increased risk of CVD among women with a BMI <25

The study concludes that the women with a BMI<25 that have a weight loss of 1 unit in BMI from pre-pregnancy until 18 mo postpartum may have an increased risk of CVD, and this association foremost seen in the group of women that also gain below the IOM guidelines in weight during pregnancy.

The mechanisms are somewhat discussed in the discussion, but I wonder if the authors have further looked into this association and tried to find an explanation? Are the numbers sufficient to stratify BMI further, i.e. separate underweight and normal weight - could it be that this relationship could be modified by BMI further, i.e. is there a stronger relationship among underweight for example. There could also be unmeasured confounding, which the authors also discussed somewhat. Further, as the follow-up time is 16 years there could also be other factors /weight loss/wegiht gain during this period of time that could play an important role. Perhaps this could be discussed even further in the discussion.

*Unfortunately, we do not have enough data to stratify on prepregnancy BMI groups further, which is why we only have stratified in 2 groups, i.e. underweight/normal-weight and overweight/obese. We have added a sentence about unknown confounding and reverse causation to the discussion of the findings for weight loss and risk of CVD among women gaining below the IOM recommendations to make this more explicit. Further, we have added a sentence to the general discussion on the potential loss of fat-free mass and the adverse effects related to that as well as in a sentence about the possible influence of weight change during the 16 years of follow-up to the general discussion of limitations.*

*Line 311-313: “Thus, it is possible that some sort of unknown confounding generates the association, or that reverse causality may be in play with subclinical CVD inducing weight loss, followed by subsequent clinically manifest CVD.”*

*Line 319-323: “Low fat-free mass [46,47] and, in elderly people [48], loss of fat-free mass is associated with increased mortality. It is likely that normal/underweight women who lost weight from before pregnancy to 18 months postpartum had lost not only fat mass but also fat-free mass, with an adverse effect on their cardiovascular health.”*

*Line 348-349: “Also, we are unaware of the mother´s weight patterns during the follow-up period which may affect the associations.”*

Minor

Abstract - New weight gain is a bit hard to understand when reading only the abstract, hence adding the time-period to define new gainers would be beneficial

*We have added the time period for the new gain to the abstract.*

*Line 14-16: “These risks were similar whether weight gain presented postpartum weight retention or a new gain from 6 months to 18 months postpartum, …”*

Figure 2 - This figure is beautiful and clearly describes the different weight patterns, however it would benefit much by adding different colors to the lines.

*Thank you! We have added colors to the figure, please see Figure 2.*

**Reviewer #2:**

This manuscript examines weight changes among the Danish National Birth Cohort from pre-pregnancy to 18 months postpartum and the associated with the incidence of cardiovascular disease and hypertension. Cardiovascular disease is one of the leading causes of death among women around the world and pregnancy and the postpartum period represent a critical time for identifying women who may be at increased risk and for implementing risk reduction strategies. A challenge for clinicians and researchers working in this field is the male-centric nature of many of our available risk identification tools. In this manuscript the authors identify and explore female-specific risk factors for hypertension and cardiovascular disease. This is valuable work that adds significant information to this field of research.

*Thank you very much for this supportive comment.*

The methods used in the analysis are robust, appropriate and well described.

With regards to outcomes, the secondary analysis including self-identified hypertension diagnosis strengthens the results, considering that hypertension is often a primary care diagnosis.

While examining only codes related to hypertension, ischemic heart disease and stroke is valid, I wonder whether a broader definition of cardiovascular disease, such as the composite outcome included in the CANHEART Study, would strengthen the results of this analysis. Could the authors elaborate on their reasoning for including their specific set of outcomes?

[Tu JV, C.A., Donovan LR, Ko DT, Booth GL, Tu K, et al., The Cardiovascular Health in Ambulatory Care Research Team (CANHEART): Using Big Data to Measure and Improve Cardiovascular Health and Healthcare Services. Circulation: Cardiovascular Quality and Outcomes, 2015. 8(2): p. 204-212.]

*Our combined endpoint: fatal and non-fatal Ischemic heart disease (encompassing acute myocardial infarction, unstable angina, chronic ischemic heart disease (CABG, PCI) and stroke (cerebrovascular disease, ischemic or hemorrhagic stroke, Transient ischemic attack) was pre-specified, which we - for that reason - find most appropriate to report. Hypertension represents a risk factor for our CV outcome, and we find it highly relevant to report this for the reader to see the size of these events. Hypertension could be viewed as an intermediate variable.*

*We acknowledge that it could be relevant to include heart failure (HF), peripheral vascular disease (PVD) and atrial fibrillation (AF), but both HF and PVD would be relatively rare is this population, and AF may reflect a very different pathology, and we are not sure this would be relevant. Diabetes which also often results in atherosclerotic disease is also another pathology that could have been relevant in the same manner as hypertension. However, we think that development of diabetes and subsequent CV outcomes could be relevant for a separate paper.*

*Due to the rarity of HF, PVD, AF in this population and diabetes mellitus as a separate risk factor for CVD, we respectfully decline their inclusion in the analysis. The focus of the paper was to examine if weight gain would lead to hypertension and atherosclerotic events. This was with the assumption that increase in weight would increase the risk of hypertension, and abdominal fat may result in metabolic syndrome. However, we could not make a Registry-based definition of metabolic syndrome.*

With regards to covariates, could the authors specify whether the intensity of exercise was considered?

*Unfortunately, we do not have information about intensity of exercise.*

When controlling for pregnancy complications, was gestational hypertension considered? Or only preeclampsia?

*The potential pathways between weight changes and CVD are complex, and pregnancy complications are early markers for later CVD. We therefore omitted adjusting for these factors except for the following 3 complications that all have their specific potential effect on GWG:*

- *Preeclampsia: It is well-known that preeclampsia may lead to extreme weight gain due to edema. Such women may therefore have high GWG and postpartum weight patterns driven by this*
- *Preterm birth: Women with preterm birth do not have a full pregnancy period to gain weight, which may affect their GWG and postpartum weight patterns.*
- *Diabetes mellitus or GDM: Women suffering from diabetes are held in very restrictive dietary regimes during pregnancy which may affect their GWG.*

*We therefore adjusted for these 3 categories of pregnancy complications in our models.*

Did the authors consider adjusting for exposure to pregnancy complications in pregnancies prior to the index pregnancy?

*We did not have the information on pregnancy complications in prior pregnancies.*

Were other placental disorders, such as abruption, considered?

*No, we did not consider this in the analyses. Please see above.*

The authors have appropriately addressed the limitations of this work and highlighted the strengths.

The conclusions appear to be supported by the results presented.

This study addresses an important issue and provides information that may help to improve women's long term health and primary preventative care.

Thank you very much for these comments.

**Reviewer #3:**

This is a well-conducted study on the association between maternal weight change from prepregnancy to 18 months postpartum and subsequent risk of hypertension and cardiovascular disease using a large national birth cohort. The study design, datasets, statistical methods and analyses, and presentation (tables and figures) and interpretation of results are mostly adequate.

*Thank you.*

However, there are still a few issues needing attention.

1) In statistical methods, the authors said "to address the problem of missing data in covariates, we used multiple imputation". It seems that all the analyses with missing data were automatically done with multiple imputation. However, there are always some bias when imputing data. Researcher normally do this in two steps, using the complete data for the formal analyses and then use imputed data for sensitivity analyses. Can authors please follow this good practice if possible? Quite a few variables with 20-30% missing data are still in the analyses which is a bit worrying. The other way to get around this is to create a dummy category of 'unknown' for those missing data so all the data can be analysed.

*We did the multiple imputation analyses as our main analyses, because MI analyses are more likely to give less biased estimates than the complete case analyses due to weaker assumptions than for the complete case analyses. Further, differences between MI analyses and complete cases analyses, will primarily elucidate the missingness mechanism in the complete case analyses. However, we did a complete cases analysis of our main analyses presented in Table 2 as part of the initial analyses and the results were in the same direction and approximate magnitude as those from the MI analyses, but the confidence intervals were wider. We decided to only present the MI analyses, as these should correct biases that may arise in complete cases analyses. The complete case analyses are mentioned in the statistical method section.*

*[*Sterne, Jonathan A C, Ian R White, John B Carlin, Michael Spratt, Patrick Royston, Michael G Kenward, Angela M Wood, and James R Carpenter. “Multiple Imputation for Missing Data in Epidemiological and Clinical Research: Potential and Pitfalls.” BMJ 338, no. jun29_1 (June 2009): b2393. <https://doi.org/10.1136/bmj.b2393>.*]*

*Line 195-197: “As part of our initial analyses, we carried out a complete case analysis of our main exposure and observed results of same direction and approximate magnitude as those presented.”*

2) Table 1 should appear in supplementary information as just recommendations.

*We have moved Table 1 to the Supplemental material and revised the text accordingly.*

3) Table 2. All the categorical variables should be presented with count (percentage) rather than just percentage.

*We have added the counts to the already presented percentage, please see Table 1.*

4) Table 3. All the 95% CI for rates and HRs should be (xx, yy) not (xx; yy). Please replace ";" with ",". Also, in the footnote, please mention the statistical methods used to derive these rates and HRs. The same also applies to Table 4 and 5.

*We have revised the tables accordingly.*

5) How many deaths were recorded during the 16-years follow up? How were they treated in the analyses? This should be discussed as there is potentially a competing risk from death when the outcomes are hypertension and CVD.

*Women were considered at risk from the date of second postpartum interview (approximately 18 months postpartum) until the time of diagnosis, emigration, death, or end of follow-up (September 10, 2018), whichever came first. Thus, women who died were censored in the Cox regression analyses when they died. Of the 47,966 women in the study population, 336 women were censored due to death during follow-up in the hypertension analyses and 348 in the CVD analyses. We believe that competing risk from this number of deaths is limited. Further, we were interested in studying associations rather than the cumulative incidences, and the hazard ratios of the Cox regression model are less susceptible to bias from competing risks than cumulative incidences. We have stated the number of deaths during follow-up in the manuscript.*

*Line 159-162: “Women were considered at risk from the date of second postpartum interview (approximately 18 months postpartum) until the time of diagnosis, emigration, death (n=336 in the hypertension analyses, n=348 in the CVD analyses), or end of follow-up (September 10, 2018), whichever came first.”*
