# Supplementary material for: Maternal weight change from prepregnancy to 18 months postpartum and subsequent risk of hypertension and cardiovascular disease in Danish women: A cohort study
Source: PLoS Med. 2021 Apr 2;18(4):e1003486. doi: 10.1371/journal.pmed.1003486 (PMC8051762; doi:10.1371/journal.pmed.1003486)
Supplement: Attachment — Submitted filename: Response to editor comment 20210215.docx [file pmed.1003486.s011.docx]

Response to editor comment:

"Thank you for making amendments to the paper to address the query about p values for interaction with prepregnancy BMI.

However, I'm afraid we are still struggling to make sense of the p values for interaction quoted in the abstract and those given at line 253, for example ("... interaction with prepregnancy BMI was observed, p=0.048 and p=0.03 (Table 3)").

Please explain where the p values for interaction are quoted in the results section or supplementary files. If you wish to quote these in the abstract, we suggest adding them in the footnotes to table 3, for example.

Reply:

Dear Richard Turner,

We understand your concern about this. In fact, the first two interactions in the abstract are from Table 2 (0.01 and 0.03), only the last one (0.03) is from Table 3. This means that an interaction in Table 3 (0.048) is not listed in the abstract, only in the text, but this is not an important finding and mainly covered in the findings from Table 2. We have now clarified this by listing the p-values for the presented interactions in the text and as foot notes in Table 2 and Table 3.

We have kept the parenthesis (Interaction by prepregnancy BMI, P=0.01, 0.03, and 0.03, respectively) in the abstract, but we could leave it out in the abstract, if you prefer, as these values are presented clearly in the main text and tables now.

Best regards,

Helene Kirkegaard
